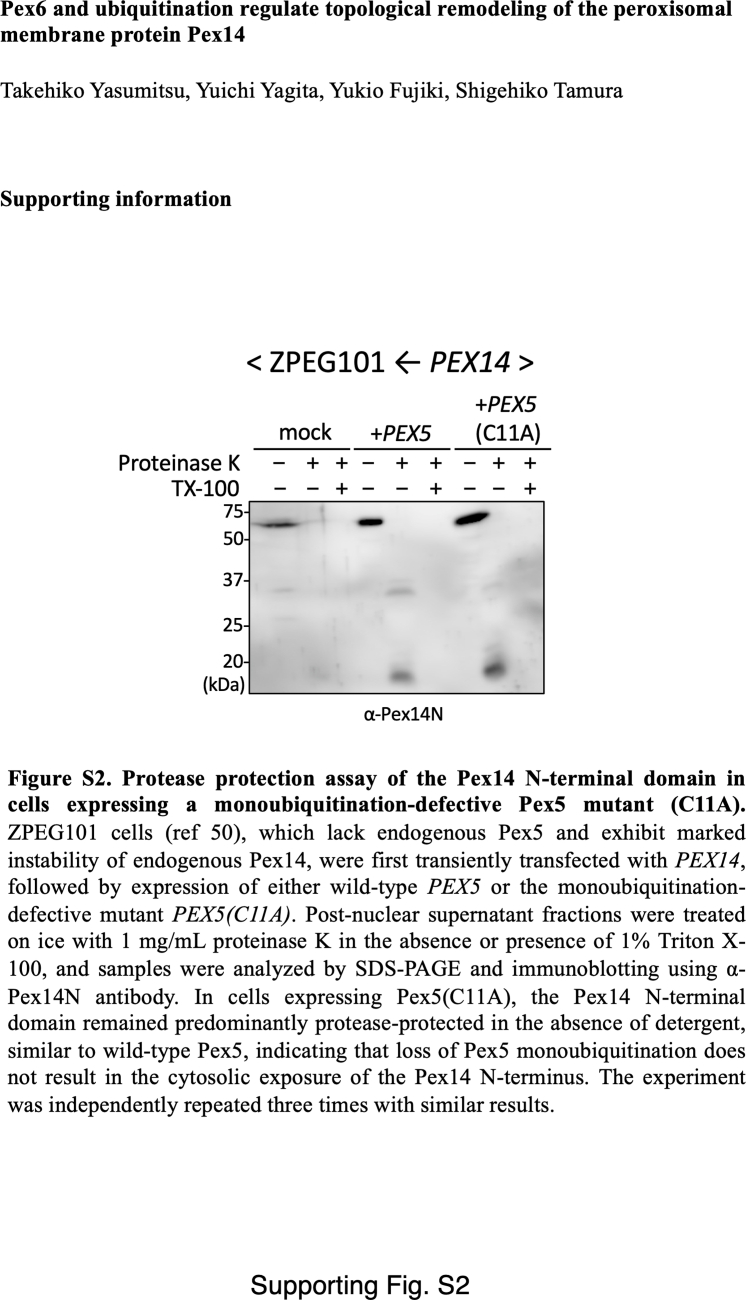# Pex6 and ubiquitination regulate topological remodeling of the peroxisomal membrane protein Pex14

**DOI:** 10.1016/j.jbc.2026.111203

**Published:** 2026-01-23

**Authors:** Takehiko Yasumitsu, Yuichi Yagita, Yukio Fujiki, Shigehiko Tamura

**Affiliations:** 1 Graduate School of Systems Life Sciences, Kyushu University, Fukuoka, Japan; 2 Faculty of Arts and Science, Kyushu University, Fukuoka, Japan; 3 Institute for Advanced Study, Kyushu University, Fukuoka, Japan; 4 Graduate School of Science, University of Hyogo, Harima Science Garden City, Hyogo, Japan

**Keywords:** peroxisome, protein translocation, AAA+ ATPase, ubiquitination, membrane topology

## Abstract

Pex14 is a membrane peroxin that plays a central role in matrix protein import by mediating the docking of the cytosolic receptor Pex5, which delivers cargo harboring a peroxisome targeting signal 1. We previously reported the crystal structure of the conserved N-terminal domain of Pex14, which harbors the primary binding site for Pex5. However, the mechanistic contribution of this domain to the import process, particularly regarding its membrane orientation, remains unclear. In this study, we investigated the role of the AAA+ ATPase peroxin Pex6 in regulating the membrane topology of the Pex14 N-terminal domain. By a combination of immunofluorescence microscopy and protease protection assays, we show that the orientation of the Pex14 N terminus is dynamically modulated in an ATP- and ubiquitination-dependent topological remodeling. Under normal culture conditions, the N-terminal domain of Pex14 is oriented toward the peroxisomal lumen. Deficiency of Pex6 or its membrane-recruiting partner Pex26, as well as pharmacological inhibition of AAA+ ATPases, resulted in a marked topological remodeling, exposing the Pex14 N terminus to the cytoplasm. Conversely, inhibition of ubiquitin activation using MLN-7243 prevented this reorientation, likely by blocking Pex5 ubiquitination and its subsequent extraction from the membrane. These findings support a model in which Pex14 undergoes reversible, ATP-dependent topological remodeling during Pex5 recycling, functioning as a molecular reset mechanism for the docking–translocation complex. Our study reveals an additional mechanism of regulation in peroxisomal protein import and highlights the coordinated roles of Pex6 and Pex5 ubiquitination in maintaining the structural organization of the translocation machinery.

Peroxisomes are essential organelles involved in a wide range of cellular metabolic processes, including fatty acid β-oxidation, detoxification of reactive oxygen species, and the maintenance of redox homeostasis (1, 2). Proper localization of matrix proteins to the peroxisomal lumen is critical for these functions, and defects in the import machinery result in severe metabolic disorders, such as Zellweger spectrum disorders (3, 4, 5, 6). Unlike other organelles, peroxisomes are capable of importing fully folded and even oligomeric proteins through a unique, ATP-independent translocation mechanism, which remains incompletely understood (5).

Most peroxisomal matrix proteins contain a C-terminal peroxisomal targeting signal type 1 (PTS1), typically represented by the tripeptide Ser-Lys-Leu (7). The cytosolic receptor Pex5 recognizes the PTS1 motif and shuttles cargo proteins to the peroxisomal membrane, where it interacts with the docking–translocation complex, composed primarily of Pex14 and Pex13 (8, 9). Among these components, Pex14 serves as the principal membrane anchor by binding multiple WxxxF/Y motifs within Pex5 through its N-terminal domain (10).

We earlier resolved the crystal structure of the conserved N-terminal domain encompassing amino acid residues 25 to 70 of Pex14, which adopts a three-helix bundle that specifically binds the WxxxF/Y motifs of Pex5 (11). This structural insight elucidated the molecular basis of the Pex14–Pex5 interaction and shed light on the organization of the docking–translocation complex. Although the precise membrane topology of the Pex14 N-terminal domain was not initially established, recent protease protection studies have reported that this region faces the peroxisomal matrix, supporting an N_in_–C_out_ topology (12, 13). However, it remains unclear whether this orientation is static or undergoes dynamic rearrangement during the protein import cycle. Notably, Pex5 has been shown to transiently integrate into the peroxisomal membrane during translocation, adopting a dynamic topology that spans both the cytosol and the peroxisomal membrane (14, 15). Based on this transient membrane interaction, it is plausible that the membrane orientation of the Pex14 N-terminal domain is also subject to dynamic topological remodeling in concert with the topological state of Pex5. In particular, Pex5 ubiquitination and ATPase activity of AAA- ATPase peroxins may serve as triggers for such topological remodeling, coupling cargo release to structural resetting of the import machinery. In contrast, the C-terminal domain of Pex14 is consistently oriented toward the cytoplasm and subject to regulation *via* phosphorylation (16, 17, 18). Additional structural features, including coiled-coil domains and GxxxG/AxxxA motifs, may contribute to Pex14 oligomerization and remodeling of the docking–translocation complex (16).

Following cargo release into the peroxisomal lumen, Pex5 is exported back to the cytosol through a tightly regulated, ATP-dependent process that is initiated by monoubiquitination at a conserved N-terminal cysteine residue (19, 20, 21, 22). In yeast, this modification is catalyzed by the E2 enzyme Pex4 and the RING-type E3 ligases Pex2, Pex10, and Pex12 (23). In mammals, where Pex4 is absent, members of the UbcH5 family serve as E2 enzymes, with Pex10 functioning as the primary E3 ligase. The RING peroxins may also contribute to the formation of a translocation pore through which the unstructured N terminus of Pex5 is returned to the cytosol (24). Monoubiquitinated Pex5 is extracted from the membrane by the heterohexameric type II AAA+ ATPase complex Pex1–Pex6 (19, 20, 25, 26), which is anchored to the peroxisomal membrane by Pex26 in mammalian cells (27). ATP hydrolysis at the D2 domains of Pex1–Pex6 has been proposed to provide the energy necessary for the extraction of a substrate through the central channel of the complex, with ubiquitinated Pex5 representing a plausible candidate (28). Notably, although cargo import is ATP independent, Pex5 recycling strictly depends on ATP hydrolysis (17), highlighting a mechanistic coordination between ubiquitin signaling and ATPase-driven receptor extraction.

Functional impairment of Pex1 or Pex6 leads to the accumulation of ubiquitinated Pex5 on the peroxisomal membrane, often triggering pexophagy (29, 30, 31). Under these conditions, both monoubiquitinated and polyubiquitinated forms of Pex5 have been detected (32, 33). Monoubiquitination facilitates Pex5 extraction and prevents its polyubiquitination and subsequent degradation (34). Furthermore, monoubiquitination at the conserved cysteine residue functions as a redox-sensitive regulatory switch. Oxidative stress inhibits this modification, thereby delaying matrix protein import (35, 36). These findings highlight the dual regulatory role of cysteine monoubiquitination in promoting Pex5 recycling and modulating import efficiency in response to redox signals. Despite growing insight into the mechanisms of Pex5 ubiquitination and extraction, whether and how these processes affect the structural organization of the docking–translocation complex, particularly regarding the membrane topology of Pex14, remains poorly understood. In particular, it has not been directly investigated whether Pex14 undergoes topological reorientation in response to Pex5 ubiquitination and Pex6 activity.

In this study, we investigated whether the N-terminal domain of Pex14 undergoes ATP- and ubiquitination-dependent topological remodeling during matrix protein import. Using immunofluorescence microscopy, protease protection assays, and a *PEX6*-deficient cellular model, we show that the membrane orientation of Pex14 is dynamically remodeled during the import cycle. Our findings reveal a previously unrecognized regulatory mechanism in peroxisomal protein import, in which Pex6 activity and ubiquitinated Pex5 cooperatively modulate the membrane topology of Pex14, thereby coordinating Pex5 recycling with structural resetting of the import apparatus.

## Results

### Pex6 and Pex26 are specifically required for maintaining the membrane topology of the Pex14 N-terminal domain

To explore the possibility that the membrane topology of the Pex14 N-terminal domain may undergo dynamic changes in coordination with matrix protein import, we examined its orientation in control and in peroxin-deficient human fibroblasts under semi- and full-permeabilization conditions. Cells were fixed under standard culture conditions and permeabilized with either digitonin, which selectively solubilizes the plasma membrane while preserving peroxisomal membrane integrity, or Triton X-100, which disrupts all membranes. Immunofluorescence staining was performed using antibodies specific to the N-terminal (α-Pex14N) or C-terminal (α-Pex14C) domains of Pex14.

**Figure 1:**
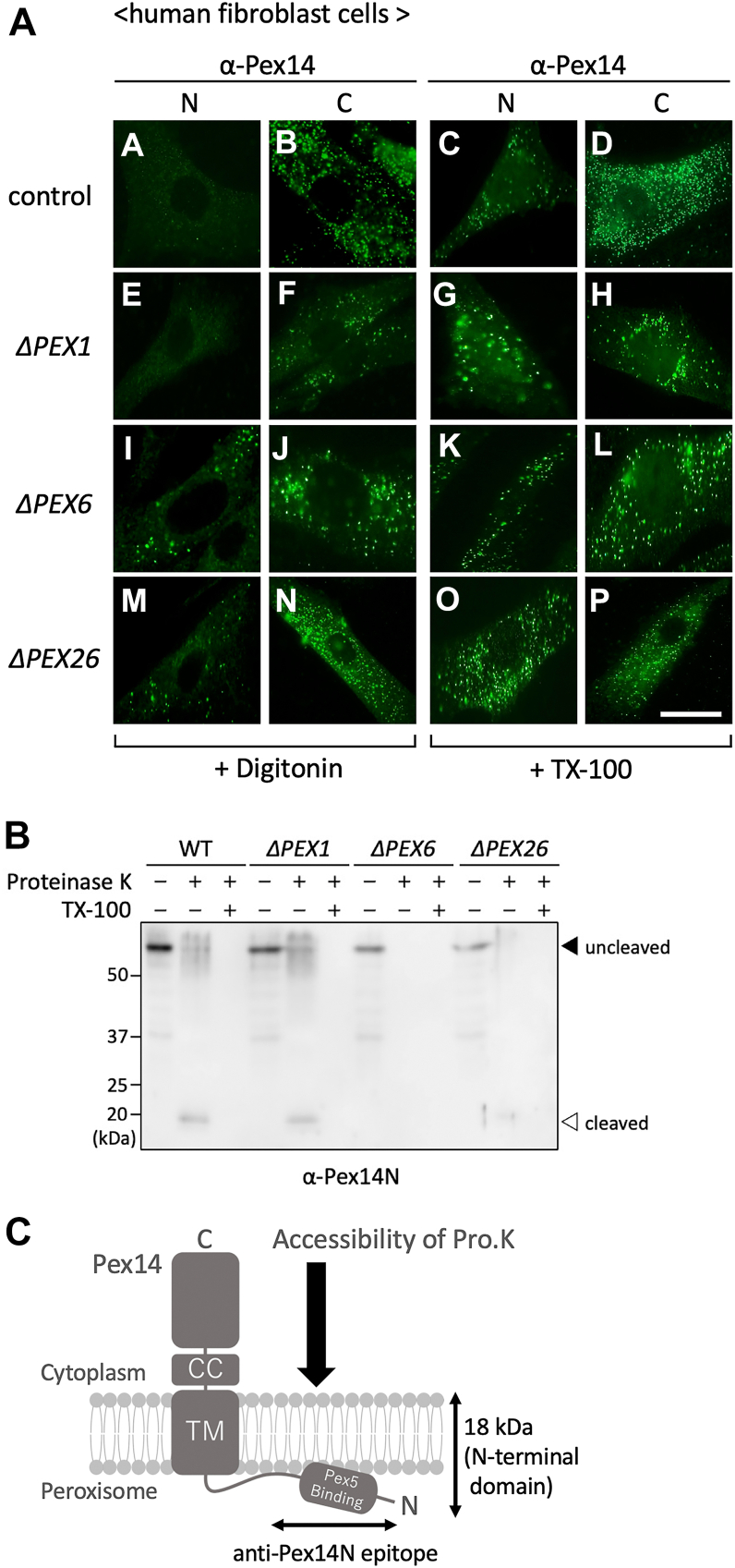
**Pex6 and Pex26 are required to maintain the proper membrane topology of the Pex14 N-terminal domain.***A,* immunofluorescence analysis of the membrane orientation of the N-terminal (N) and C-terminal (C) domains of Pex14 in control, Δ*PEX1* (*pex1*), Δ*PEX6* (*pex6*), and Δ*PEX26* (*pex26*) human fibroblasts. Cells were selectively permeabilized with 60 μg/ml digitonin (*left panels*), which preserves peroxisomal membrane integrity while allowing access to cytoplasmically exposed epitopes, or fully permeabilized with 1% Triton X-100 (*right panels*). Samples were stained with antibodies directed against the N-terminal domain (α-Pex14N; *panels a, c, e, g, i, k, m, and o*) or the C-terminal domain (α-Pex14C; *panels b, d, f, h, j, l, n, and p*) of Pex14. The scale bar represents 20 μm. *B,* protease protection assay to assess the membrane topology of the Pex14 N-terminal domain. Postnuclear supernatants (PNSs) from control and mutant fibroblasts were treated with 1 mg/ml proteinase K for 30 min on ice in the absence or presence of 1% Triton X-100. Samples were analyzed by SDS-PAGE and immunoblotting using α-Pex14N antibody. A *solid arrowhead* indicates full-length Pex14; an *open arrowhead* denotes an ∼18-kDa protease–resistant fragment corresponding to the membrane-protected N-terminal domain. *C,* schematic diagram of the protease accessibility assay. Under normal conditions, the Pex14 N-terminal domain, which includes the Pex5-binding region, is inserted into the peroxisomal membrane and oriented toward the matrix, rendering it inaccessible to cytosolic protease (Pro. K). In Δ*PEX6* (*pex6*) or Δ*PEX26* (*pex26*) cells, this domain becomes misoriented and exposed to the cytosol, resulting in proteolytic degradation. The epitope recognized by the α-Pex14N antibody is indicated in the schematic. All experiments shown in *A* and *B* were independently repeated at least three times with similar results, and representative images and immunoblots are shown. CC, coiled-coil domain; TM, transmembrane domain.

**Figure S1:**
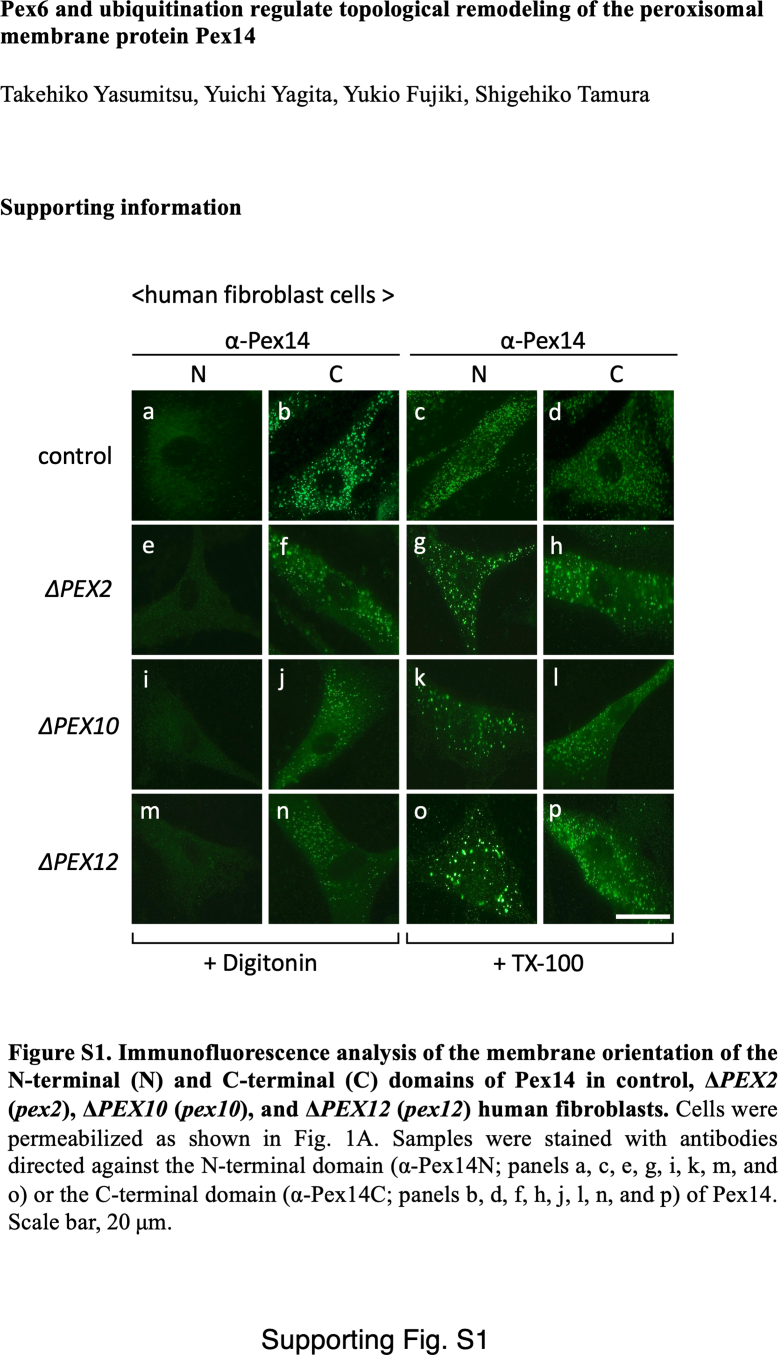


Under full permeabilization (Triton X-100), both α-Pex14N and α-Pex14C signals were consistently detected as punctate structures across all cell lines tested, confirming epitope accessibility (Fi 1*A*, panels c, d, g, h, k, l, o, and p). In contrast, digitonin-permeabilized control fibroblasts showed punctate staining only with α-Pex14C, whereas α-Pex14N signals were *undetectable* (Fig. 1*A*, panels a and b). This staining pattern, consistent with previous reports, indicates that under normal conditions, the C terminus of Pex14 is exposed to the cytosol and the N terminus is oriented toward the peroxisomal lumen or embedded within the membrane. A similar staining pattern was observed in *PEX1*-, *PEX2-*, *PEX10-*, and *PEX12-*deficient fibroblasts (Fig. 1*A*, panels e and f; Fig. S1), suggesting that these peroxins are dispensable for maintaining the membrane topology of Pex14. In contrast, fibroblasts deficient in *PEX6* or *PEX26* exhibited distinct punctate staining for both α-Pex14N and α-Pex14C antibodies following digitonin treatment (Fig. 1*A*, panels i, j, m, and n), indicating aberrant cytoplasmic exposure of the Pex14 N terminus. This staining pattern indicates that the N-terminal domain of Pex14 is abnormally exposed to the cytoplasm in these mutant cells. Such topological alteration was not observed in fibroblasts lacking other peroxins, including Pex1, Pex2, Pex10, and Pex12, thereby highlighting the specific requirement for Pex6 and Pex26 in maintaining the correct membrane orientation of Pex14.

Together, these results show that the proper lumen-facing orientation of the Pex14 N-terminal membrane–embedded domain depends specifically on Pex6 and Pex26. Notably, *PEX1*-deficient cells retained normal topology, even though Pex1 forms a complex with Pex6 as part of the AAA+ ATPase machinery, indicating that Pex1 is not required for controlling Pex14 membrane topology. Instead, Pex6 and its membrane anchor Pex26 appear to play a more direct role. The cytoplasmic exposure of the Pex14 N terminus in the absence of Pex6 or Pex26 implicates a Pex6- and ATP-dependent mechanism that couples Pex14 topological remodeling to dynamic reorganization of the docking–translocation complex during the matrix protein translocation.

### Protease protection assay confirms topological alterations in the Pex14 N-terminal domain

To biochemically validate the topological changes observed by immunofluorescence microscopy, protease protection assays were performed on postnuclear supernatants (PNSs) from control and mutant fibroblasts, followed by immunoblotting with an α-Pex14N antibody. In control cells, an ∼18-kDa protease–resistant fragment of Pex14 was detected following proteinase K treatment (Fig. 1*B*, WT). This fragment was completely digested in the presence of Triton X-100, suggesting that the N-terminal domain of Pex14 was partially protected from digestion because of its insertion into the lipid bilayer or translocation across the peroxisomal membrane. Upon addition of Triton X-100, the protected fragment was completely digested, confirming that the observed protection results from membrane embedding rather than its intrinsic resistance to proteolysis. A similar digestion pattern was observed in *PEX1*-deficient cells (Δ*PEX1*), consistent with the immunofluorescence findings, suggesting that Pex1 is not required for maintaining the native membrane topology of Pex14. In contrast, no protease-protected fragment was detected in *PEX6*-deficient cells, indicating that the N-terminal domain of Pex14 is fully exposed to the cytoplasm and therefore entirely susceptible to proteolytic digestion. In *PEX26*-deficient cells, the ∼18 kDa protected fragment was faintly detected, suggesting a reduction in membrane-associated protection (Fig. 1*B*, Δ*PEX6* and Δ*PEX26*). These biochemical results corroborate the immunofluorescence data and strengthen the conclusion that Pex6, and to a lesser extent Pex26, is essential for maintaining the lumen-facing orientation of the Pex14 N-terminal domain. The protease accessibility model shown in Figure 1*C* illustrates that under normal conditions, the N-terminal domain of Pex14 is embedded within the peroxisomal membrane and oriented toward the matrix, rendering it inaccessible to externally added proteases. In the absence of Pex6, this topology is disrupted, resulting in cytoplasmic exposure of the N-terminal domain and its susceptibility to proteolytic cleavage, whereas loss of Pex26 causes only a mild effect on this topology. Together, these findings indicate that Pex6, which is recruited to the peroxisomal membrane by Pex26, is essential for maintaining the membrane-protected topology of the Pex14 N-terminal domain.

### Functional analysis of Pex6 Walker motif mutants reveals ATPase-dependent topological regulation of Pex14

**Figure 2:**
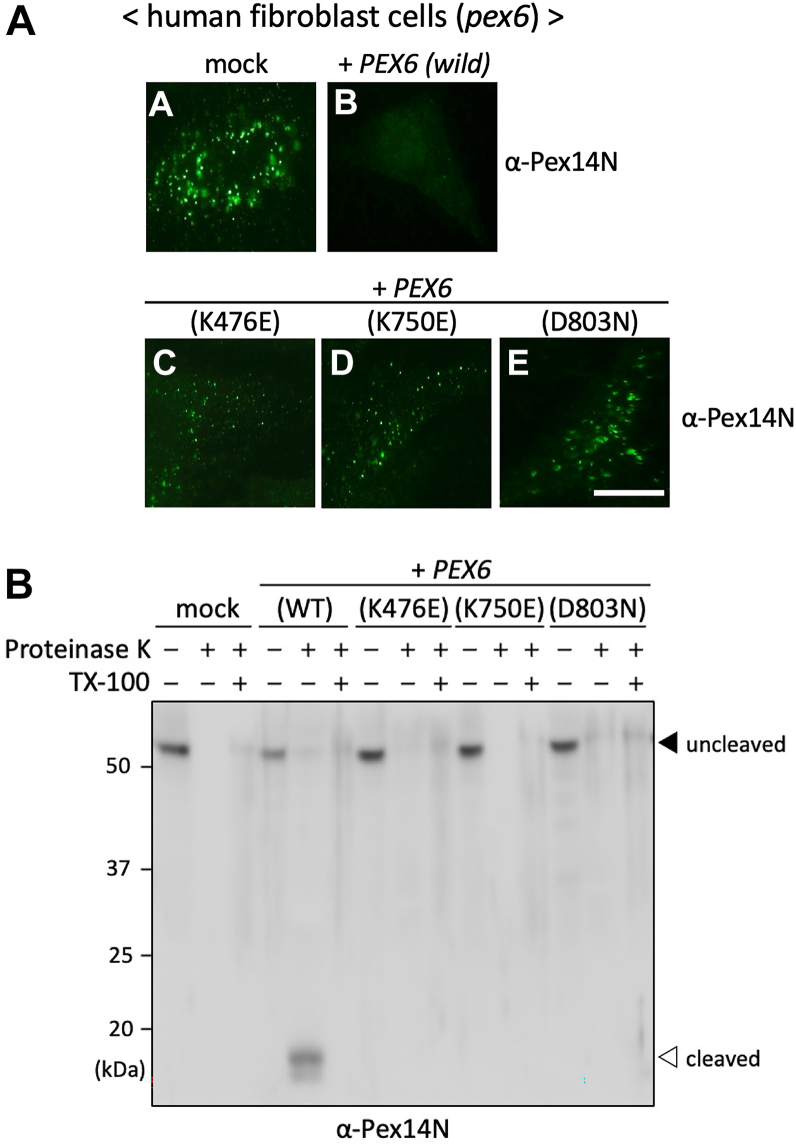
**ATPase activity of Pex6 is required for topological reorientation of the Pex14 N-terminal domain.***A,* immunofluorescence analysis of the Pex14 N-terminal domain in Δ*PEX6* (*pex6*) human fibroblasts transiently transfected with WT or Walker motif mutant Pex6 constructs. Cells were permeabilized with 60 μg/ml digitonin and stained with the antibody against the N-terminal domain of Pex14 (α-Pex14N; *panels a–e*). Expression of WT Pex6 (+*PEX6*) restored the membrane-protected localization of the N-terminal domain, as indicated by the absence of detectable α-Pex14N signal (*panel b*). In contrast, cells expressing Walker A mutants K476E (D1) or K750E (D2), or the Walker B mutant D803N (D2), failed to re-establish this topology, thus exhibiting strong punctate α-Pex14N staining apparently comparable to mock-transfected cells (*panels a, c–e*). The scale bar represents 20 μm. *B,* protease protection assay assessing the membrane orientation of the Pex14 N-terminal domain in the same transfected cells as shown in (*A*). Postnuclear supernatants (PNSs) were treated on ice with 1 mg/ml proteinase K for 30 min in the absence or the presence of 1% Triton X-100. Samples were separated by SDS-PAGE and analyzed by immunoblotting using α-Pex14N antibody. A *solid arrowhead* denotes full-length Pex14, and an *open arrowhead* indicates the ∼18 kDa protease–protected fragment corresponding to the N-terminal domain, either embedded in the membrane or lumen-facing orientation. All experiments were independently repeated at least three times with similar results.

To dissect the roles of ATP binding and hydrolysis in Pex6-dependent regulation of Pex14 membrane topology, we introduced point mutations in the conserved Walker motifs of Pex6. These included K476E in the Walker A motif of the D1 domain (A1), K750E in the Walker A motif of the D2 domain (A2), and D803N in the Walker B motif of the D2 domain (B2). The Walker B motif of the D1 domain was not targeted for mutagenesis, as it lacks the canonical aspartate and glutamate residues required for ATP hydrolysis. These mutant constructs were separately expressed in *PEX6*-deficient human fibroblasts, and the membrane orientation of the Pex14 N-terminal domain was assessed by immunofluorescence microscopy and protease protection assays. Expression of WT Pex6 restored the membrane-protected orientation of the Pex14 N-terminal domain, as shown by the absence of punctate α-Pex14N signals after digitonin treatment (Fig. 2*A*, panel b). In sharp contrast, cells expressing any of the Walker motif mutants retained punctate α-Pex14N staining, indicating a failure to promote topological reorientation of the Pex14 N-terminal domain (Fig. 2*A*, panels c–e). These results suggest that both ATP binding and hydrolysis are essential for Pex6-mediated translocation of the Pex14 N terminus across the peroxisomal membrane.

To further confirm these observations, we also performed protease protection assays (Fig. 2*B*). In cells expressing WT Pex6, an ∼18-kDa protease–protected fragment of Pex14 was detected following proteinase K treatment, indicating successful translocation of the N-terminal domain into a membrane-protected compartment. In contrast, no protected fragment was detected in cells separately expressing the K476E, K750E, and D803N mutants, consistent with cytosolic exposure of the N-terminal domain and a failure to restore the membrane-protected topology of the Pex14 N-terminal domain. Together, these results suggest that both membrane translocation and reorientation of the Pex14 N-terminal domain require ATP binding to the Walker A motifs and ATP hydrolysis by the Walker B motif of the Pex6 D2 domain. These findings support a model in which Pex6 acts as an ATP-dependent regulator of Pex14 topology and are consistent with previous reports highlighting the essential role of the Walker motifs in peroxisomal matrix protein import (37).

### ATP depletion promotes cytoplasmic exposure of the Pex14 N-terminal domain

**Figure 3:**
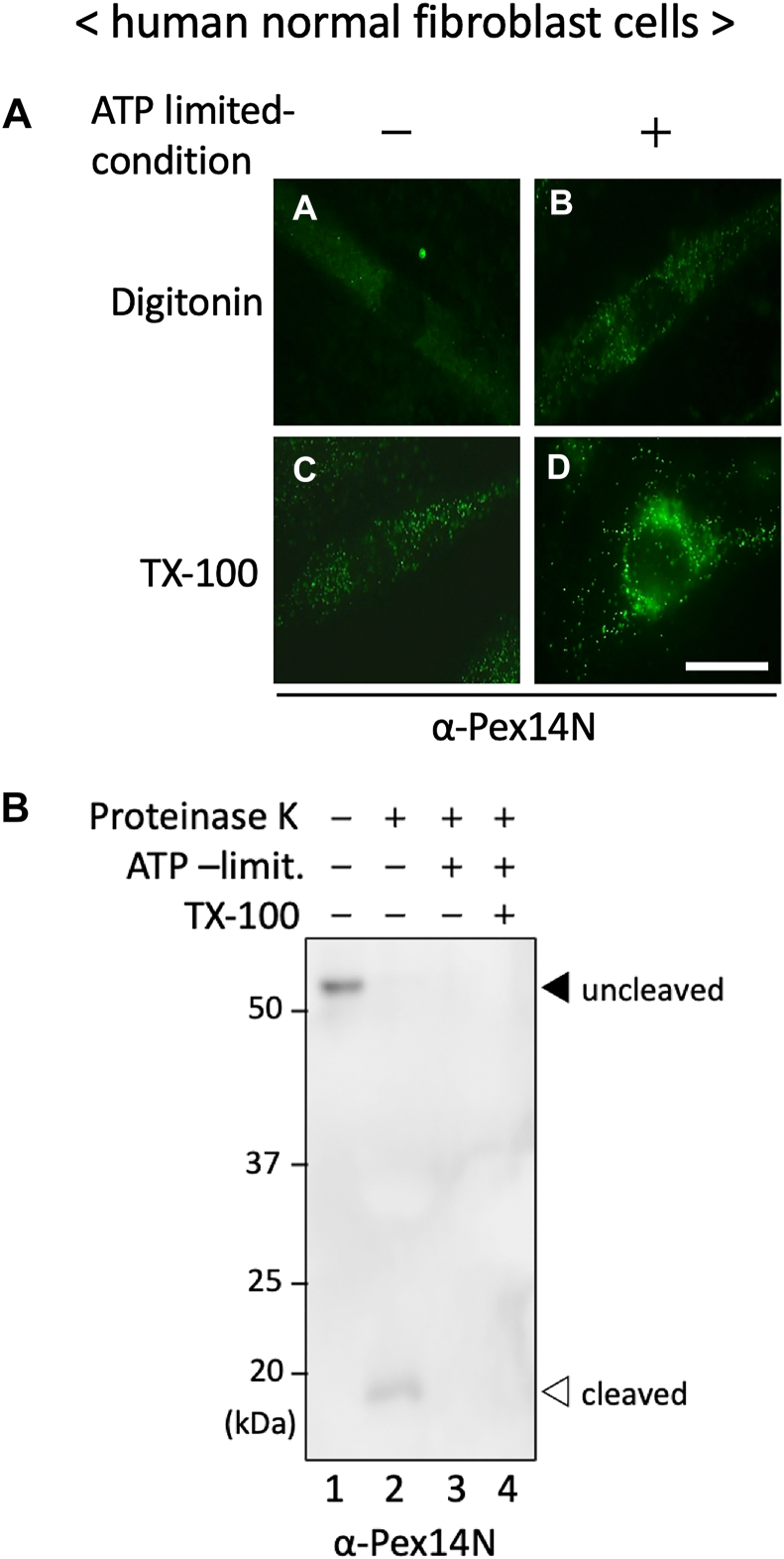
**ATP is required to maintain the membrane-protected topology of the Pex14 N-terminal domain.***A,* immunofluorescence analysis of the Pex14 N-terminal domain in human fibroblasts under ATP-depleted conditions. Cells were incubated for 6 h at 37 °C in ATP-depletion medium (DMEM without glucose, supplemented with 10 mM sodium azide, 10 mM sodium fluoride, and 50 mM 2-deoxy-d-glucose) and then selectively permeabilized with 60 μg/ml digitonin (*panels a, b*) or fully permeabilized with 1% Triton X-100 (*panels c, d*). Samples were stained with α-Pex14N antibody. The scale bar represents 20 μm. *B,* protease protection assay to assess the membrane topology of the Pex14 N-terminal domain under ATP-depleted conditions. PNS fractions were treated on ice with 1 mg/ml proteinase K for 30 min in the absence or the presence of 1% Triton X-100. Samples were analyzed by SDS-PAGE and immunoblotting using α-Pex14N antibody. A *solid arrowhead* denotes full-length Pex14, and an *open arrowhead* indicates the ∼18-kDa protease–resistant fragment corresponding to the membrane-embedded N-terminal domain. All experiments were independently repeated at least three times with comparable results. DMEM, Dulbecco's modified Eagle's medium; PNS, postnuclear supernatant.

Pex5 is known to accumulate on the peroxisomal membrane under ATP-depleted conditions (29), and our previous work demonstrated that Pex26 acts as a membrane anchor that spatially coordinates the ATPase activity of Pex6 toward the Pex14–Pex5 complex within the functional docking–translocation complex (38). To determine whether ATP availability affects the membrane orientation of the Pex14 N-terminal domain, we assessed its accessibility by immunofluorescence staining and protease protection assays following cellular ATP depletion. Digitonin-permeabilized cells showed no α-Pex14N signal, consistent with luminal orientation or membrane embedding of the Pex14 N-terminal domain under normal conditions. In contrast, ATP depletion induced a distinct punctate α-Pex14N staining pattern, suggesting that the N-terminal domain became exposed to the cytoplasmic face (Fig. 3*A*, panels a and b). Notably, α-Pex14N signals were clearly detected under Triton X-100 permeabilization regardless of ATP availability, indicating that once membrane barriers are removed, the N-terminal epitope is equally accessible to antibody detection (Fig. 3*A*, panels c and d). Consistently, protease protection assays showed that the ∼18-kDa protease–protected fragment of Pex14 was lost upon ATP depletion (Fig. 3*B*). These findings are in good agreement with those observed in *PEX6*- and *PEX26*-deficient cells (Fig. 1*B*) and support the conclusion that ATP hydrolysis is required for maintaining the membrane-protected topology of the Pex14 N-terminal domain.

### Inhibition of AAA+ ATPases alters the membrane topology of Pex14

**Figure 4:**
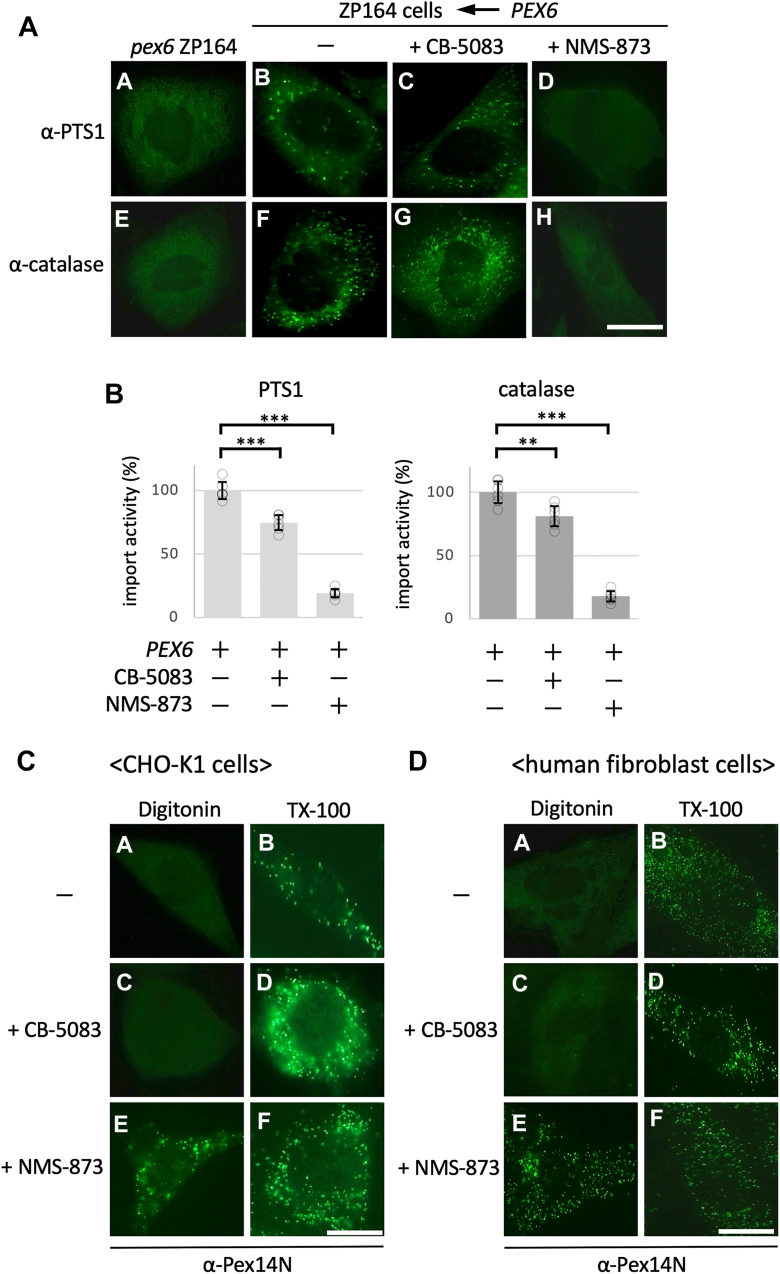
**Pharmacological inhibition of AAA+ ATPases impairs peroxisomal matrix protein import and alters the membrane topology of the Pex14 N-terminal domain.***A,* immunofluorescence analysis of peroxisomal matrix protein import in ZP164 cells (*PEX6*-deficient CHO cells) transfected with WT *PEX6*. Cells were treated with DMSO (vehicle control; *panels b, f*), 100 nM CB-5083 (panels c, g), or 100 nM NMS-873 (panels d, h) for 72 h, fixed, permeabilized with 1% Triton X-100, and stained with antibodies against PTS1-containing proteins (*panels a–d*) and catalase (*panels e–h*). Mock-transfected cells (*pex6* ZP164, *panels a, e*) served as a negative control. The scale bar represents 10 μm. *B,* quantitative analysis of import activity shown in (*A*). Import efficiency was calculated as the percentage of Pex14-positive cells exhibiting punctate signals for PTS1-containing proteins or catalase. Data represent mean ± SD from three independent biological experiments, each quantified from six technical measurements (n = 6). Individual data points are shown. Statistical significance was determined using unpaired two-tailed *t* tests (∗∗*p* < 0.01, ∗∗∗*p* < 0.001). *C* and *D,* immunofluorescence analysis of the membrane orientation of the Pex14 N-terminal domain in CHO-K1 cells (*C*) and human fibroblasts (*D*) following ATPase inhibition. Cells were treated with DMSO (vehicle control; panels a, b), 50 nM CB-5083 (*panels c, d*), or 10 μM NMS-873 (*panels e, f*) for 72 h and then subjected to selective permeabilization with 60 μg/ml digitonin (*left panels*) or full permeabilization with 1% Triton X-100 (*right panels*). Samples were stained with α-Pex14N antibody. The scale bars represent 10 μm (*C*) and 20 μm (*D*). CHO, Chinese hamster ovary cell line; DMSO, dimethyl sulfoxide; PTS1, peroxisomal targeting signal type 1.

To assess the dependence of Pex14 membrane topology on AAA+ ATPase activity, we employed two small-molecule inhibitors targeting this enzyme family. CB-5083 and NMS-873 are known to target valosin-containing protein (p97), a AAA+ ATPase that shares structural homology with Pex6. CB-5083 acts as a competitive inhibitor at the ATP-binding site (IC_50_ = 11 nM), whereas NMS-873 functions allosterically (IC_50_ = 30 nM) (39, 40, 41). Although these compounds have not been defined for direct inhibition of Pex6, they serve as useful chemical probes to evaluate the involvement of AAA+ ATPases, including Pex6, in the regulation of peroxisomal protein import. To evaluate the effect of these compounds on Pex6-mediated matrix protein import, we expressed WT Pex6 in the *PEX6*-deficient Chinese hamster ovary (CHO) cell line ZP164 and examined the peroxisomal localization of matrix proteins in the presence or absence of each inhibitor. As shown in Figure 4*A*, expression of Pex6 restored peroxisomal targeting of both endogenous PTS1-harboring proteins and catalase in untreated cells (Fig. 4*A*, panels b and f). Treatment with CB-5083 moderately impaired the import of both the PTS1-targeted protein and catalase (Fig. 4*A*, c and g), whereas treatment with NMS-873 markedly inhibited their peroxisomal localization (Fig. 4*A*, d and h). Quantitative analysis confirmed a significant reduction in import efficiency by NMS-873 treatment (Fig. 4*B*).

To determine whether these effects were associated with alterations in Pex14 membrane topology, we next performed immunofluorescence analysis in CHO-K1 cells and human fibroblasts treated with respective compounds. In control cells, digitonin permeabilization did not reveal α-Pex14N staining, indicating a membrane-protected or lumen-facing orientation (Fig. 4, *C* and *D*, panels a). In contrast, NMS-873 treatment resulted in punctate α-Pex14N staining (Fig. 4, *C* and *D*, panels e), suggesting cytoplasmic exposure of the N-terminal domain. Treatment with CB-5083 gave rise to a barely detectable increase in signal intensity (Fig. 4, *C* and *D*, panels c). Under full permeabilization conditions with Triton X-100, a punctate α-Pex14N staining pattern was observed in all samples (Fig. 4*C*, panels b, d, f), indicating that the epitope remained detectable even after inhibitor treatment. Taken together, these results indicate that pharmacological inhibition of AAA+ ATPases, particularly with NMS-873, disrupts the membrane-protected topology of the Pex14 N-terminal domain. These findings support a model in which ATP hydrolysis by Pex6 is essential for maintaining the correct membrane orientation of Pex14 during peroxisomal matrix protein import.

### Ubiquitination of Pex5 promotes cytoplasmic orientation of the Pex14 N-terminal domain and Pex5

**Figure 5:**
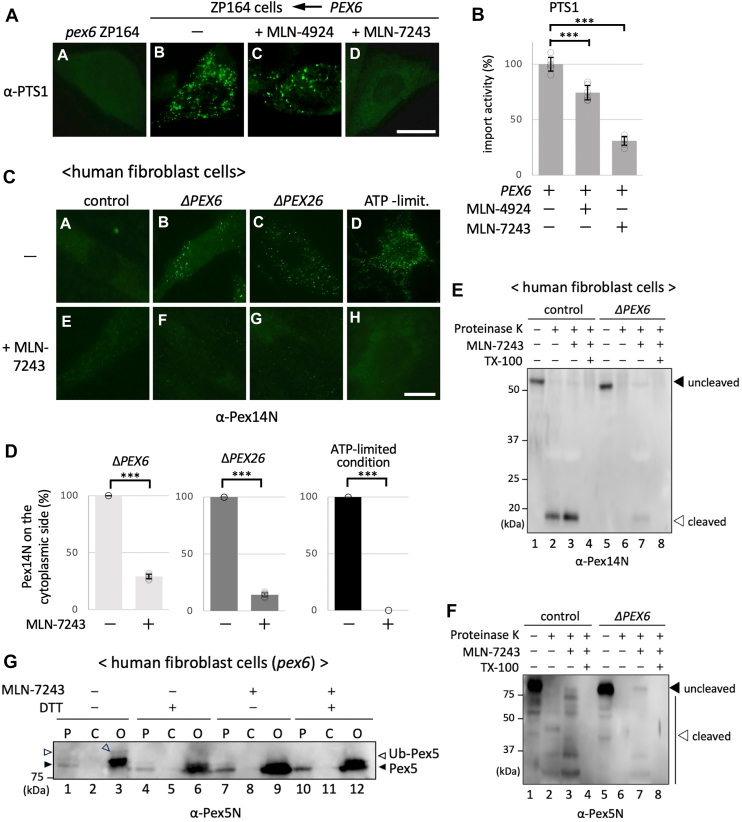
**Ubiquitination of Pex5 promotes cytoplasmic exposure of the Pex14 N-terminal domain and regulates its membrane topology.***A,* immunofluorescence analysis of peroxisomal matrix protein import in ZP164 cells (*PEX6*-deficient CHO cells) transfected with either mock (*panel a*) or WT *PEX6* (*panels b–d*). Cells were treated for 6 h with DMSO (vehicle control; *panel b*), 10 μM MLN-4924 (panel c), or 10 μM MLN-7243 (*panel d*), then fixed, permeabilized with 1% Triton X-100, and stained with an antibody against the PTS1 epitope. The scale bar represents 10 μm. *B,* quantification of import activity shown in (*A*), calculated as the percentage of Pex14-positive cells exhibiting punctate PTS1 signals. Data represent mean ± SD from three independent biological experiments, each quantified using six technical replicates (n = 6). Individual data points are shown. Statistical significance was determined by unpaired two-tailed *t* tests (∗∗∗*p* < 0.001). *C,* immunofluorescence analysis of the membrane orientation of the Pex14 N-terminal domain in control, Δ*PEX6*, Δ*PEX26*, and ATP-depleted human fibroblasts. Cells were treated for 6 h with DMSO (vehicle control; *panels a–d*) or 10 μM MLN-7243 (panels e–h), selectively permeabilized with 60 μg/ml digitonin, and stained with α-Pex14N antibody. The scale bar represents 20 μm. *D,* quantitative analysis of cytoplasmic exposure of the Pex14 N-terminal domain shown in (*C*). Data represent the percentage of cells with detectable α-Pex14N signal following digitonin permeabilization. Results are shown as mean ± SD from three biological replicates, each assessed in six technical replicates (n = 6). ∗∗∗*p* < 0.001 by unpaired two-tailed *t* tests. *E,* protease protection assay assessing the membrane topology of the Pex14 N-terminal domain in control and Δ*PEX6* fibroblasts with or without MLN-7243 treatment. PNS fractions were incubated on ice with 1 mg/ml proteinase K for 30 min in the absence or the presence of 1% Triton X-100. Samples were analyzed by SDS-PAGE and immunoblotting using α-Pex14N. *Solid and open arrowheads* indicate full-length Pex14 and the ∼18 kDa protease–resistant fragment, respectively. *F,* protease protection assay of Pex5 under the same conditions as (*E*). Full-length Pex5 (*solid arrowhead*) and partial digestion products (*open arrowhead*) were detected in control cells and increased following MLN-7243 treatment. *G,* immunoblot analysis of Pex5 ubiquitination in Δ*PEX6* fibroblasts. PNS (P) fractions were prepared from *PEX6*-deficient human fibroblast cells treated with or without 1 μM MLN-7243 for 6 h. Samples were incubated with 10 mM *N*-ethylmaleimide (NEM) to preserve ubiquitin conjugates, fractionated by centrifugation into the cytosolic (C) and organelle (O) fractions, and subjected to SDS-PAGE. Where indicated, samples were further treated with 50 mM DTT to reduce cysteine-linked ubiquitin conjugates. An aliquot of the C fraction equivalent to the P fraction and seven aliquots of the O fraction were loaded. Immunoblotting was performed using an anti-Pex5N antibody. *Open and closed arrowheads* indicate monoubiquitinated and unmodified Pex5, respectively (50). CHO, Chinese hamster ovary cell line; DMSO, dimethyl sulfoxide; PNS, postnuclear supernatant; PTS1, peroxisomal targeting signal type 1.

Previous studies have shown that ubiquitinated Pex5 accumulates on peroxisomal membranes in cells lacking AAA-type peroxins, suggesting a role for ubiquitination in facilitating the recycling of Pex5 from the peroxisome to the cytosol (31). We earlier identified AWP1, a ubiquitin-binding regulator of NF-κB signaling, as a factor that selectively recognizes cysteine-monoubiquitinated Pex5 and promotes its extraction through interaction with Pex6 (20). Supporting these findings, Skowyra *et al.* (13). recently reported that MLN-7243, an inhibitor of the E1 ubiquitin–activating enzyme UBA1, significantly impairs the import of PTS1-containing proteins. To investigate whether Pex5 ubiquitination contributes to the regulation of Pex14 membrane topology, we initially evaluated the effect of MLN-7243 on Pex6-mediated restoration of matrix protein import in the *PEX6*-deficient CHO cell line ZP164. As shown in Figure 5*A*, expression of WT Pex6 restored peroxisomal targeting of PTS1-containing proteins, which was markedly suppressed by MLN-7243 treatment (Fig. 5*A*, panels b and d). In contrast, MLN-4924, an inhibitor of neural precursor cell–expressed developmentally downregulated protein 8–activating enzyme, had only a modest effect (Fig. 5*A*, panel c). Quantitative analysis confirmed a significant reduction in import efficiency following MLN-7243 treatment as compared with either MLN-4924 or vehicle control (Fig. 5*B*), highlighting the critical role of ubiquitination in peroxisomal matrix protein import.

We next assessed the effect of MLN-7243 on the membrane orientation of the Pex14 N-terminal domain in control fibroblasts and *PEX6-* or *PEX26-*deficient fibroblasts and control fibroblasts depleted of ATP. In control fibroblasts, α-Pex14N staining was not detected following digitonin permeabilization under either untreated or MLN-7243-treated conditions, indicating that the N-terminal domain remained embedded in the membrane or oriented toward the lumen (Fig. 5*C*, panels a and e). By contrast, *PEX6-* and *PEX26-*deficient cells, as well as ATP-depleted cells, exhibited punctate α-Pex14N staining under untreated conditions (Fig. 5*C*, panels b–d), consistent with cytoplasmic exposure of the N-terminal domain. Notably, punctate α-Pex14N staining was no longer detectable following MLN-7243 treatment (Fig. 5*C*, panels f–h). Quantitative analysis revealed that MLN-7243 induced reorientation of the Pex14 N-terminal domain toward the lumen in more than 70% of cells across all experimental conditions (Fig. 5*D*). To corroborate these observations biochemically, we performed protease protection assays in control and *PEX6*-deficient fibroblasts. In control cells, the ∼18 kDa protease–resistant fragment corresponding to the Pex14 N-terminal domain was consistently detected under both untreated and MLN-7243-treated conditions (Fig. 5*E*, lanes 2 and 3), indicating stable membrane protection. In contrast, this protected fragment was absent in untreated *PEX6*-deficient cells but was faintly recovered following MLN-7243 treatment (Fig. 5*E*, lanes 6 and 7), suggesting partial restoration of the lumen-facing topology of the Pex14 N terminus.

We also examined the membrane association of Pex5 by performing protease protection assays. In control cells, partial digestion products of Pex5 were detected, consistent with partial membrane insertion and protection from proteolysis (Fig. 5*F*). Treatment with MLN-7243 increased the abundance of both full-length and partially digested forms of Pex5, suggesting enhanced membrane association. In untreated *PEX6*-deficient cells, full-length and partially digested Pex5 bands were barely detectable (Fig. 5*F*, lane 6), indicating complete cytosolic exposure. Remarkably, MLN-7243 treatment restored both the full-length form and partial digestion products of Pex5 in these cells (Fig. 5*F*, lane 7). Notably, this restoration coincided with the reappearance of the ∼18-kDa Pex14 fragment, implying a coordinated topological shift of both Pex14 and Pex5. To directly confirm that MLN-7243 inhibits Pex5 monoubiquitination under the same conditions that promote reorientation of the Pex14 N-terminal domain and restore membrane association of Pex5, we performed immunoblot analysis of PNS fraction from *PEX6*-deficient fibroblasts. Samples were fractionated into the cytosolic and organelle fractions and analyzed under both reducing and nonreducing conditions using an anti-Pex5 antibody. In untreated *PEX6*-deficient cells, a slower-migrating band consistent with monoubiquitinated Pex5 (Ub-Pex5) (19, 20) was detected in the organelle fraction (Fig. 5*G*, lane 3), along with unmodified Pex5. The Ub-Pex5 band was sensitive to DTT and disappeared under reducing conditions, confirming that it represents a cysteine-linked ubiquitinated form. Treatment with MLN-7243 abolished the Ub-Pex5 band without affecting unmodified Pex5 (Fig. 5*G*, lane 9), indicating that the inhibitor effectively blocked Pex5 monoubiquitination *in vivo*.

Collectively, these findings suggest that monoubiquitination of Pex5 acts as a regulatory signal that facilitates the cytoplasmic orientation of both the Pex14 N-terminal domain and Pex5 during peroxisomal matrix protein import. However, the potential involvement of polyubiquitination or other modifications cannot be excluded and should be further investigated. In the absence of ubiquitination, the N-terminal domain of Pex14 remains oriented toward the lumen, implying that reorientation toward the cytoplasmic face is tightly coupled to the presence of ubiquitinated Pex5. These results further support a model in which ATP-dependent disassembly of the Pex14–Pex5 complex by Pex6 is followed by topological remodeling of the Pex14 N-terminal domain, thereby resetting the import apparatus for subsequent rounds of matrix protein translocation.

## Discussion

This study reports that the membrane topology of the N-terminal domain of Pex14 is dynamically regulated during peroxisomal matrix protein import and that this regulation is tightly coordinated with both the ATPase activity of Pex6 and the ubiquitination status of Pex5. We propose that after cargo translocation, the peroxisomal import machinery undergoes a resetting phase, during which the Pex14 N-terminal domain reorients from the cytoplasm back to the lumen to prepare for the next import cycle. Specifically, we show that Pex6 is required for maintaining the canonical lumen-facing orientation of the Pex14 N-terminal domain, whereas its membrane-anchoring partner Pex26 contributes to this process. In the absence of Pex6, this domain becomes reoriented toward the cytoplasm, indicating a failure in this proposed resetting phase of the docking–translocation complex, whereas loss of Pex26 results in a more modest effect. The essential role of ATPase activity in this topological regulation is supported by our mutational analysis of Pex6. Expression of Walker motif mutants defective in either ATP binding or hydrolysis failed to restore the lumen-facing orientation of the Pex14 N-terminal domain in *PEX6*-deficient cells, indicating that both functions are required for the topological reorientation of Pex14. Consistent with this ATP-dependent regulatory model, we previously demonstrated that WT FLAG–Pex26/Pex6/Pex1 complexes do not coimmunoprecipitate with Pex14, whereas ATPase-defective Pex6 mutants (K750E and D803N) selectively coprecipitate with Pex14 (38). These findings support the idea that ATP hydrolysis by Pex6 regulates its interaction with Pex14 and further underscore the essential role of ATPase activity in controlling Pex14 membrane topology. Pharmacological inhibition of AAA-type ATPases with NMS-873 induced a phenotype resembling that observed in *PEX6*-deficient cells, further supporting the conclusion that ATP hydrolysis is essential for topological rearrangement of Pex14. Consistently, ATP depletion alone induced cytoplasmic exposure of the Pex14 N-terminal domain, highlighting the energy dependence of this membrane configuration.

The second major finding is that Pex5 ubiquitination appears to contribute to the cytoplasmic exposure of both Pex5 and the Pex14 N-terminal domain. Inhibition of ubiquitin activation by MLN-7243 prevented the topological remodeling typically observed in *PEX6*- and *PEX26*-deficient cells. This interpretation was further supported by the appearance of protease-protected fragments of Pex14 under the same experimental conditions, suggesting that monoubiquitinated Pex5 may act as a regulatory signal that promotes reorientation of the Pex14 N-terminal domain toward the cytoplasmic face. In addition, using a monoubiquitination-defective Pex5 mutant (Pex5-C11A), we confirmed that the Pex14 N-terminal domain remains protected on the luminal side under these conditions (Fig. S2), supporting a specific role of monoubiquitination in this process. However, other potential ubiquitination sites or additional regulatory mechanisms have not yet been examined. Collectively, these results suggest that Pex5 ubiquitination influences the membrane topology of the Pex14 N-terminal domain. They also raise the possibility that Pex6 facilitates topological remodeling of Pex14 by dissociating the interaction between ubiquitinated Pex5 and the N-terminal domain of Pex14 in an ATP-dependent manner, although the molecular basis of this dissociation remains unclear, thereby allowing its reorientation. Importantly, our data do not support a model in which Pex6 itself directly provides the driving force for reorientation of the Pex14 N-terminal domain toward the lumen. Rather, we propose that the primary role of Pex6 is to dissociate ubiquitinated Pex5 from Pex14 in an ATP-dependent manner, thereby permitting the N-terminal domain of Pex14 to disengage from its cytosol-oriented state. The subsequent reorientation of the Pex14 N-terminal domain toward the luminal side may instead be driven by additional components of the import machinery, potentially including Pex13, which forms the core of the translocation channel together with Pex14. Elucidating the molecular mechanism underlying this resetting step of Pex14 topology remains an important subject for future investigation.

**Figure 6:**
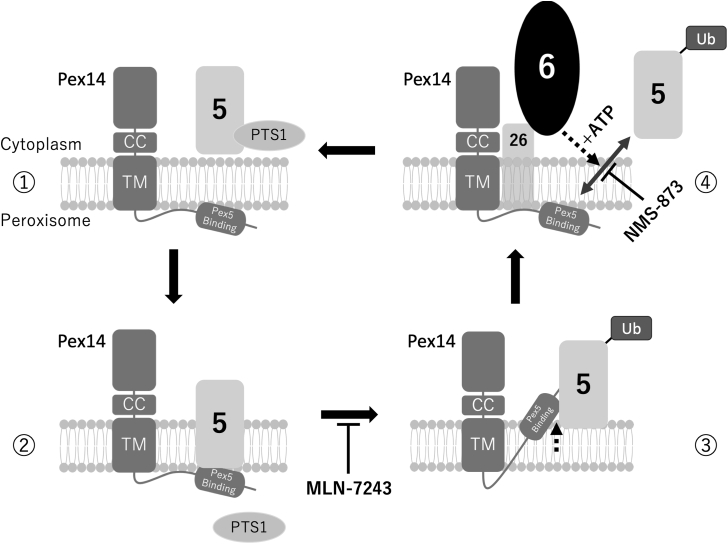
**Proposed model for ATP- and ubiquitination-dependent topological conversion of the Pex14 N-terminal domain.** Under basal conditions, Pex14 is integrated into the peroxisomal membrane with its N-terminal domain embedded and oriented toward the lumen, where it facilitates matrix protein translocation. Upon cargo delivery, the cytosolic receptor Pex5, carrying a PTS1-containing protein, docks onto the Pex14-containing docking–translocation complex (step 1). Following cargo release, Pex5 undergoes monoubiquitination at its N-terminal cysteine residue, which is proposed to trigger a topological rearrangement that reorients the N-terminal domain of Pex14 toward the cytoplasmic side (steps 2 and 3). This cytosol-oriented conformation may facilitate the recruitment and recycling of Pex5. The AAA+ ATPase Pex6, anchored to the membrane *via* Pex26, recognizes ubiquitinated Pex5 and extracts it from the peroxisomal membrane in an ATP hydrolysis–dependent manner (step 4). This extraction process is concurrently coupled to the restoration of the lumen-facing topology of the Pex14 N-terminal domain, effectively resetting the docking–translocation complex for subsequent rounds of protein import. Pharmacological inhibition of ubiquitination using MLN-7243 blocks the outward topological switch, whereas inhibition of ATPase activity by NMS-873 prevents the inward reorientation. Together, these findings support a model in which dynamic and reversible regulation of Pex14 topology is integral to the peroxisomal protein import cycle. CC, coiled-coil domain; PTS1, peroxisomal targeting signal type 1; TM, transmembrane domain.

On the basis of our observations, we propose a model illustrated in Figure 6. Under resting conditions, Pex14 is embedded in the peroxisomal membrane with its N-terminal domain facing the lumen, where it mediates cargo translocation by interacting with Pex5. During this process, Pex5 partially inserts into the membrane, likely through lipid interactions (14), positioning its N-terminal domain near the luminal face of Pex14, and facilitating interaction with the Pex14 N-terminal domain. Upon cargo release, cysteine-linked monoubiquitination of Pex5 appears to drive its cytosolic repositioning, which may subsequently induce a topological shift of the Pex14 N-terminal domain toward the cytoplasm. This cytosol-oriented conformation likely facilitates interaction with the Pex6–Pex26 complex, enabling ATP-dependent extraction of ubiquitinated Pex5. The extraction step is followed by reorientation of the Pex14 N-terminal domain back toward the lumen, thereby resetting the import machinery for subsequent rounds of matrix protein import. Pharmacological inhibition using MLN-7243 or NMS-873 highlights the sequential requirement of Pex5 ubiquitination and ATP hydrolysis by Pex6 in orchestrating this membrane topology remodeling process. However, the mechanism that drives the reorientation of the Pex14 N-terminal domain back toward the luminal side after the dissociation of monoubiquitinated Pex5 by Pex6 has yet to be determined.

These findings collectively suggest that Pex14 is not a static structural scaffold but rather a dynamic component of the docking–translocation complex whose membrane topology is subject to regulated remodeling. Such topological plasticity may contribute to the coordination of cargo delivery, receptor recycling, and structural resetting of the import machinery. While this study expands our understanding of the dynamic behavior of Pex14, several mechanistic aspects remain to be clarified. It remains unclear whether the observed reorientation of Pex14 reflects complete membrane translocation of its N-terminal domain across the membrane or instead involves more subtle rearrangements, such as partial reinsertion or local conformational rearrangements. These potential mechanisms may involve regulatory events that are not readily detectable under the experimental conditions used in this study. Moreover, the contributions of additional components of the docking–translocation complex, including Pex1 and the RING peroxins, to the regulation of Pex14 topology, either through direct interaction or through post-translational modifications, remain to be fully elucidated. In addition, whether Pex6 directly promotes the dissociation of ubiquitinated Pex5 from the N-terminal domain of Pex14 to induce topological remodeling has not been clearly established and may require further investigation. Notably, deficiency of Pex1 does not result in cytoplasmic exposure of the Pex14 N-terminal domain, indicating that Pex1 is not essential for maintaining its proper membrane topology. It should be noted that the *PEX1*-deficient fibroblasts used in this study have been reported to harbor a nonsense mutation introducing a premature stop codon at Gln261, together with additional splicing abnormalities (42). One reported splice variant lacks exon 6, resulting in an in-frame internal deletion (Ile414–Leu453), which may retain partial functionality or the ability to associate with Pex6. In addition, Pex6 localizes predominantly to peroxisomes, whereas Pex1 is present in both cytosolic and peroxisomes (37). Thus, it is possible that not all cellular pools of Pex1 and Pex6 exist as fully functional complexes, which may contribute to the distinct phenotypes observed in *PEX1*- and *PEX6*-deficient cells. Although Pex1 assembles into a heterohexameric complex with Pex6, the specific contribution of Pex1 itself to the regulation of Pex14 topology, either independently or in coordination with Pex6, remains to be clarified (28). In parallel, while our findings suggest that monoubiquitination of Pex5 promotes topological remodeling of the Pex14 N-terminal domain, our analysis using the monoubiquitination-defective Pex5-C11A mutant indicates that loss of Pex5 monoubiquitination alone is not sufficient to fully account for this process. Therefore, additional studies employing mutants that selectively perturb different ubiquitination states as well as ubiquitin chain–specific probes will be necessary to determine whether monoubiquitination alone is both necessary and sufficient for Pex14 topological remodeling or whether polyubiquitination and other post-translational modifications may also contribute to this process.

In conclusion, this study identifies a previously unrecognized mechanism by which ATP hydrolysis by Pex6 and ubiquitination of Pex5 cooperatively regulate the membrane orientation of the Pex14 N-terminal domain. This mechanism coordinates energy-dependent receptor recycling with structural resetting of the import machinery, providing new insight into the dynamic regulation of peroxisomal matrix protein import.

## Experimental procedures

### Antibodies

We used rabbit polyclonal antibodies against the N-terminal (Pex14N) and C-terminal (Pex14C) domains of rat Pex14, as previously described (17, 43). Additional rabbit antisera were employed against the PTS1 peptide (10), human catalase (44), and Pex5N (19).

### Cell culture, plasmids, and DNA transfection

Fibroblasts derived from Japanese Zellweger syndrome patients *pex1* CG1 (PBDE-14), *pex2* CG10 (PBDF-01), *pex10* CG7 (PBDB-01), and *pex12* CG3 (PBD3-02) were provided by Gifu University (42, 45, 46, 47). Additional fibroblast lines from Zellweger syndrome patients *pex26* CG8 (GM07371) and *pex6* CG4 (GM13267) were obtained from the Coriell Institute for Medical Research (44). Human fibroblasts were maintained in Dulbecco's modified Eagle's medium supplemented with 10% fetal calf serum at 37 °C in 5% CO_2_, as described previously (19). WT CHO-K1 cells and peroxisome-deficient CHO mutant cell lines, including *pex6* ZP164 and *pex5* ZPEG101, were cultured in Ham's F-12 medium with 10% fetal calf serum under the same conditions (37, 48, 49). Plasmids encoding Pex6 variants and Pex5 C11A mutant were described previously (19, 37). DNA transfection was performed using Lipofectamine 3000 (Invitrogen) according to the manufacturer's protocol.

### Drug treatments

AAA+ ATPase inhibitors (NMS-873 and CB-5083) and ubiquitination pathway inhibitors, including a neural precursor cell–expressed developmentally downregulated protein 8–activating enzyme inhibitor (MLN-4924) and a ubiquitin-activating enzyme inhibitor (MLN-7243), were obtained from SelleckChem. Human fibroblasts and CHO-K1 cells were incubated with 3 μM MLN-4924 (13), 10 μM NMS-873 (40, 41), or 50 nM CB-5083 (39) for 72 h at 37 °C. MLN-7243 (1 μM) was added to human fibroblast cultures for 6 h at 37 °C (13).

### ATP depletion

ATP depletion was induced by incubating human fibroblasts in glucose-free Dulbecco's modified Eagle's medium supplemented with 10 mM sodium azide, 10 mM sodium fluoride, and 50 mM 2-deoxy-d-glucose for 6 h at 37 °C (38).

### Morphological analysis

Cells were fixed with 4% paraformaldehyde, permeabilized with either 0.1% Triton X-100 or 60 μg/ml digitonin in 1% bovine serum albumin, and incubated with primary antibodies for 1 h at room temperature (10). Alexa Fluor 488–conjugated secondary antibodies (Invitrogen) were used for indirect immunofluorescence (38). Images were acquired using a Zeiss Axioscope with a Plan Apochromat 63×/1.4 numerical aperture oil-immersion objective lens. Image processing was performed using Zen software (Zeiss). Peroxisome-restoring activity of Pex6 was verified by counting PTS1- or catalase-positive cells among 100 cells in *pex6* ZP164 cells. The import efficiency of WT Pex6 was taken as 100%.

### Protease protection assay

PNS fractions were prepared from human fibroblasts and CHO cells. Protease protection was assessed by incubating 0.5 mg/ml PNS with 1 mg/ml proteinase K on ice for 30 min, as described (13, 17). The digestion was terminated by the addition of 1 mM PMSF. The digests were analyzed by SDS-PAGE, followed by immunoblotting using rabbit anti-Pex14N and anti-Pex5N antibodies.

### Immunoblotting

Proteins were transferred to polyvinylidene membranes (Bio-Rad) and probed with primary antibodies and secondary antibodies conjugated to horseradish peroxidase (GE Health Sciences) (10). Antigen–antibody complexes were visualized with an ECL Western blotting detection reagent (GE Health Sciences).

## Data availability

All data are contained within the article.

## Supporting information

This article contains supporting information.

## Conflict of interest

The authors declare that they have no conflicts of interest with the contents of this article.

## Figures and Tables

**Figure S2:**